# Topical Colloidal Silver for the Treatment of Recalcitrant Chronic Rhinosinusitis

**DOI:** 10.3389/fmicb.2018.00720

**Published:** 2018-04-11

**Authors:** Mian L. Ooi, Katharina Richter, Catherine Bennett, Luis Macias-Valle, Sarah Vreugde, Alkis J. Psaltis, Peter-John Wormald

**Affiliations:** ^1^Department of Surgery-Otolaryngology, Head and Neck Surgery, Basil Hetzel Institute for Translational Health Research, The University of Adelaide, Adelaide, SA, Australia; ^2^Adelaide Biofilm Test Facility, Sansom Institute for Health Research, University of South Australia, Adelaide, SA, Australia; ^3^Facultad Mexicana de Medicina Universidad La Salle, Department of Otolaryngology Head and Neck Surgery, Spanish Hospital of Mexico, Granada, Mexico

**Keywords:** chronic rhinosinusitis, recalcitrant, infection, antimicrobial, topical agent, safety, efficacy

## Abstract

**Background:** The management of recalcitrant chronic rhinosinusitis (CRS) is challenged by difficult-to-treat polymicrobial biofilms and multidrug resistant bacteria. This has led to the search for broad-spectrum non-antibiotic antimicrobial therapies. Colloidal silver (CS) has significant antibiofilm activity *in vitro* and *in vivo* against *S. aureus, MRSA*, and *P. aeruginosa*. However, due to the lack of scientific efficacy, it is only currently used as an alternative medicine. This is the first study looking at the safety and efficacy of CS in recalcitrant CRS.

**Methods:** Patients were included when they had previously undergone endoscopic sinus surgery and presented with signs and symptoms of sinus infection with positive bacterial cultures. Twenty-two patients completed the study. Patients were allocated to 10–14 days of culture directed oral antibiotics with twice daily saline rinses (*n* = 11) or 10 days of twice daily 0.015 mg/mL CS rinses (*n* = 11). Safety observations included pre- and post-treatment serum silver levels, University of Pennsylvania Smell Identification Test (UPSIT) and adverse event (AE) reporting. Efficacy was assessed comparing microbiology results, Lund Kennedy Scores (LKS) and symptom scores using Visual Analog Scale (VAS) and Sino-Nasal Outcome Test (SNOT-22).

**Results:** CS demonstrated good safety profile with no major adverse events, no changes in UPSIT and transient serum silver level changes in 4 patients. CS patients had 1/11 (9.09%) negative cultures, compared to 2/11 (18.18%) in the control group upon completion of the study. Whilst not statistically significant, both groups showed similar improvement in symptoms and endoscopic scores.

**Conclusion:** This study concludes that twice daily CS (0.015 mg/mL) sinonasal rinses for 10 days is safe but not superior to culture-directed oral antibiotics. Further studies including more patients and looking at longer treatment or improving the tonicity of the solution for better tolerability should be explored.

## Introduction

The management of recalcitrant chronic rhinosinusitis (CRS) is increasingly challenged by difficult-to-treat polymicrobial biofilms and multidrug resistant bacteria which antibiotics often cannot effectively eradicate. For recalcitrant patients, antibiotics often alleviate symptoms in acute exacerbations but fail to eradicate the biofilm nidus which periodically sheds planktonic organisms resulting in a relapsing and remitting course of disease (Foreman et al., 2011). This has fuelled a continuous search for broad-spectrum topical non-antibiotic anti-biofilm therapies. Topical agents allow increased concentration, localized action, less systemic side effects and lessen the risk of antibiotic resistance.

To date, numerous topical agents have been tested and although some have shown anti-biofilm activity (Chiu et al., 2008; Le et al., 2008; Alandejani et al., 2009; Jardeleza et al., 2011; Jervis-Bardy et al., 2012; Paramasivan et al., 2014; Richter et al., 2016, 2017a,b), none have been widely accepted as a treatment option in recalcitrant CRS. Recent evidence suggests that colloidal silver (CS) may be effective against bacterial biofilms. We have previously shown that CS showed significant anti-biofilm activity *in vitro* and *in vivo* against *S. aureus* (Goggin et al., 2014; Rajiv et al., 2015), and against methicillin-resistant *S. aureus* (MRSA) and *P. aeruginosa* biofilms. Spherical nanoparticles were also shown to be non-toxic in human cell culture (THP-1, Nuli-1) (Richter et al., 2017c) and safe in a sheep sinusitis model (Rajiv et al., 2015). Moreover, they were physically stable for over 6 months in storage with no observed loss in anti-biofilm activity (Richter et al., 2017c).

However, due to the lack of evidence for their efficacy, it is only currently used as an alternative medicine. This is the first study investigating the safety and efficacy of CS in recalcitrant CRS patients.

## Methods and materials

### Participants and study design

This was a prospective, open-label, single-blinded, pilot study looking at the safety and efficacy of CS sinonasal rinses in patients with recalcitrant CRS between December 2016 to July 2017. Ethics approval was granted by the Central Northern Adelaide Health Service, Ethics of Human Research Committee (TQEH/LMH/MH HREC) to conduct the trial within its network of teaching hospitals in Adelaide, Australia. All subjects gave written informed consent in accordance with the Declaration of Helsinki.

**Figure 1:**
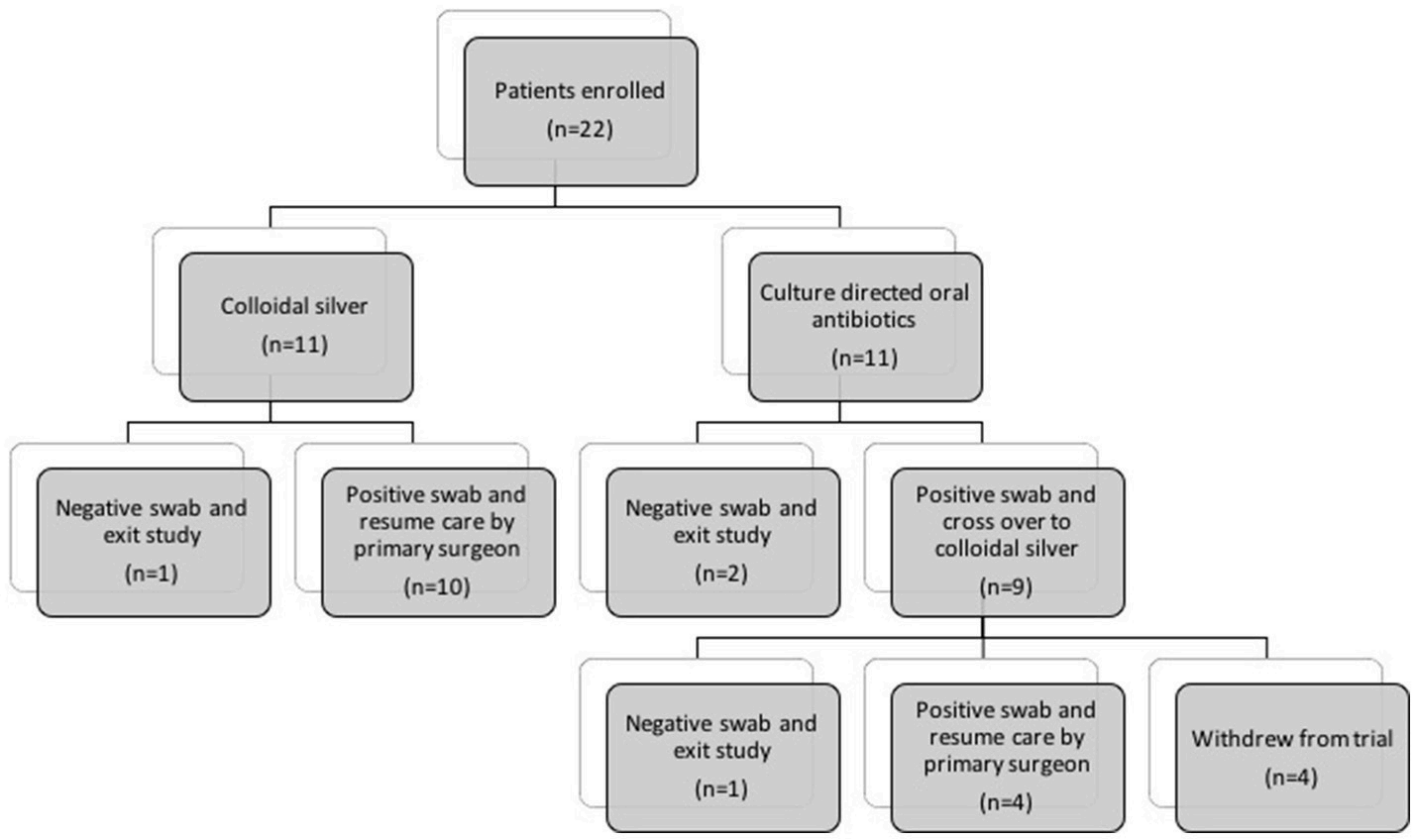
Flow diagram describing patients allocated to (1) Culture-directed oral antibiotics with twice daily saline flush (CON) and (2) Colloidal silver (CS) with twice-daily saline flush. CON, Control; CS, Colloidal silver.

A total of 22 patients were enrolled in the study (8 females, 14 males, aged 27–86). Patients were allocated to either the colloidal silver arm (CS) (*n* = 11) or control arm (CON) (*n* = 11) depending on availability of silver stock and patient's adverse reaction to culture-sensitive oral antibiotics (Figure 1). Full inclusion and exclusion criterias are outlined in Table 1. Baseline demographic and clinical characteristic are demonstrated in Table 2.

**Table 1:** Inclusion and exclusion criteria.

**Inclusion Criteria**	**Exclusion criteria**
Ongoing symptoms of CRS despite at least one trial of oral antibiotics	Allergy to silver
ESS >12 weeks prior to enrolment	Antibiotics in previous 2 weeks
Positive bacterial microbiology swab	Taking oral corticosteroids
Over 18 years of age AND able to give written informed consent	Pregnant or breastfeeding
Willing to return to this center for postoperative follow-up care	Immunocompromised

*ESS, Endoscopic sinus surgery; CRS, Chronic rhinosinusitis*.

**Table 2:** Baseline patient demographics and clinical characteristics.

	**CON (*n* = 11)**	**CS (*n* = 11)**
Age, year	61 (52–72)	60 (47–73)
Gender, male	7 (63.6%)	7 (63.6%)
History of polyposis	9 (81.82%)	8 (72.73%)
Frontal drillouts	7 (63.64%)	9 (81.82%)
Visual analog scale	38.29 (22.14–51.86)	49.72 (28.75–65)
SNOT-22 score	38.55 (23–59)	58.01 (43–75)
Lund-Kennedy score	6.82 (4–10)	8.57 (6–10)

*Data are medians (interquartile range) or numbers (%). CON, Control; CS, Colloidal silver; SNOT-22, Sino-Nasal Outcome Test-22*.

CS patients were provided with 20 sealed bottles of pre-filled 120 mL CS solution in standard nasal irrigation squeeze bottles. Patients were instructed to store these bottles away from light and in the refrigerator. Prior to use, patients were asked to warm the solution to room temperature, fill the rinse bottle to 240 mL with cooled boiled water, then perform the rinses twice daily for 10 days. Patients are to apply gentle pressure onto squeeze bottles which delivers the solution through the inner tube and out of the tip of the bottle into the nostril. CS patients were specifically instructed not to add the usual proprietary buffered salts sachets to avoid chemical interaction with the CS nanoparticles. All squeeze bottles were provided by NeilMed Pharmaceuticals (Santa Rosa, CA). If there were signs of persistent infection on endoscopic examination and a positive culture swab post-treatment, CS patients exited the study and resumed treatment based on clinical grounds.

CON patients received a 10 to 14-day course of culture-directed oral antibiotics and were instructed to perform twice daily saline rinses similar to the delivery of CS. If the patient had persistent infection on endoscopic examination and a positive culture swab at the end of treatment, they received CS.

Those taking INCs on enrolment were instructed to continue throughout the duration of the study.

### Synthesis of silver nanoparticles

Spherical silver nanoparticles were prepared as previously described (Richter et al., 2017c). Briefly, a mixture of 6.25 mL water, 1.25 mL sodium citrate (1% wt.), 1.25 mL silver nitrate (1% wt.) and 50 μl potassium iodide (300 μM) was prepared under stirring at room temperature and incubated for 5 min. This mixture was added to 237.5 mL of boiling water that included 250 μl ascorbic acid (0.1M). The colorless solution changed to yellow and finally slightly orange, indicating particle formation. The silver nanoparticles were further boiled for 1 h under reflux and stirring at 1,500 rpm. After cooling, the silver nanoparticles were characterized by UV-Vis spectrometry and transmission electron microscopy (quality control). This confirmed a spherical particle shape and size of approximately 40 nm. Silver nanoparticles were stored in amber glass flasks under dark condition at 4°C prior to utilization as a nasal rinse.

#### Efficacy assessment

Endoscopic guided sinonasal swabs were taken at every scheduled visit for microbiological evaluation. All patients completed symptoms score questionnaire at every visit, using Sino-Nasal Outcome Test-22 (SNOT-22) (Kennedy et al., 2013) (22 items, each scored from 0 to 5; total score range 0 to 110) and Visual Analog Scale (VAS) (Walker and White, 2000) (average of 6 items and an overall symptom score; each scored from 0 to 100, total score range 0 to 100). At each visit, all patients had entry and exit endoscopic videos recorded and scored by a blinded surgeon using the Lund Kennedy Score (LKS) (Lund and Kennedy, 1995; Kennedy et al., 2013) (score range, 0–20).

#### Safety assessment

All patients on CS treatment were required to have pre- and post-treatment serum silver levels and completed the University of Pennsylvania Smell Identification Test (UPSIT). If serum silver level post-treatment was above normal limits, a repeat serum silver level was performed 7 days later to confirm return to baseline. Patients were advised to report any adverse outcomes while on the study.

### Data analysis

Statistical power was calculated for the primary end-point of culture negativity post-treatment. Power analysis estimates determined a sample size of 11 patients per group would be required to achieve statistical significance (80%, *p* < 0.05) based on response rates of 25 and 90% in the control and silver groups, respectively.

All results were statistically analyzed at the completion of the study using 2-way analysis of variance (ANOVA) and student's *t*-test, with a significance value set at *p* < 0.05.

## Results

### Efficacy

#### Microbiology result

2/11 (18.18%) patients in CON group had negative swabs while 1/11 (9.09%) CS patients had negative swabs upon completion of treatment. List of pathogens treated in both cohorts are described in Table 3.

**Table 3:** Standard semi-quantitative analysis of bacterial load reported as scant, light, moderate or heavy (equivalent to 1+, 2+, 3+, or 4+) by laboratory.

**Before colloidal silver**			**After colloidal silver**
Heavy *MRSA* + Light *P. aeruginosa*			Heavy *MRSA* + Scant *P. aeroginosa*
Heavy *S. aureus*			Moderate *S. aureus*
Heavy *S. aureus* + Heavy *P. aeruginosa*			Moderate *S. aureus* + Moderate *P. aeroginosa*
Moderate *S. aureus*			Light *S. aureus* + Light *S. pneumoniae*
Scant *K. oxytoca*, Scant *H. influenza*			No growth
Heavy *H. influenza*			Heavy *S. aureus* + Light *E. cloacae* + Light *H. influenzae*
Moderate *K. oxytoca* + Moderate *P. aeruginosa*			Heavy *K. oxytoca* + Moderate *P. aeruginosa*
Light *P. aeruginosa*			Moderate *S. aureus*
Heavy *S. aureus*			Moderate *S. aureus*
Heavy *S. aureus* + Heavy *P. aeruginosa*			Heavy *S. aureus*
Heavy *S. aureus*			Heavy *S. aureus* + Moderate *M. morganii*
**Before oral antibiotics**	**Antibiotics**	**After oral antibiotics**	**After colloidal silver**
Heavy *S. aureus*	Augmentin DF	Moderate *P. aeruginosa* + Heavy *S. aureus*	Moderate *S. pneumoniae* + Moderate *S. aureus*
Moderate *P. aeruginosa*	Ciprofloxacin	Light *P. stutzeri*	Withdrew due to other commitments
Heavy *H. influenzae*	Bactrim DS	No growth	
Heavy *S. aureus*	Augmentin DF	Heavy *S. maltophilia*	No growth
Moderate *S. aureus*	Augmentin DF	No growth	
Heavy *E. coli*	Augmentin DF	Moderate *E. coli*	Withdrew due to flush discomfort
Heavy *S. aureus*	Cephalexin	Moderate *S. aureus* + Light *H. influenzae*	Withdrew due to lack of efficacy
Moderate *S. aureus*	Augmentin DF	Moderate *S. aureus* + Light *H. influenzae*	Heavy *S. aureus* + Light *E. coli*
Moderate *E. aerogenes*	Ciprofloxacin	Moderate *E. aerogenes +* Scant *S. aureus*	Withdrew due to due to external injury
Moderate *S. pneumoniae* + Scant *S. aureus*	Augmentin DF	Light *S. aureus*	Light *S. aureus*
Moderate *S. pneumoniae* + Scant *S. aureus*	Bactrim DS	Moderate *S. aureus* + Scant *Alternaria* sp.	Light *S. aureus*

*P. aeruginosa, Pseudomonas aeruginosa; MRSA, Methicllin resistant staphylococcus aureus; S. aureus, Staphylococcus aureus; H. influenza, Haemophilus Influenzae; E. cloaca, Enteroboacter cloacae; S. pneumonia, Streptococcus pneumonia; K. oxytoca, Klebsiella oxytoca; M. Morganii, Morganella Morganii; P. stutzeri, Pseudomonas stutzeri; S. maltophilia, Stenotrophomonas maltophilia; E. coli, Escherichia coli; E. aerogenes, Enterobacter aerogenes*.

#### Visual analog scale (VAS)

**Figure 2:**
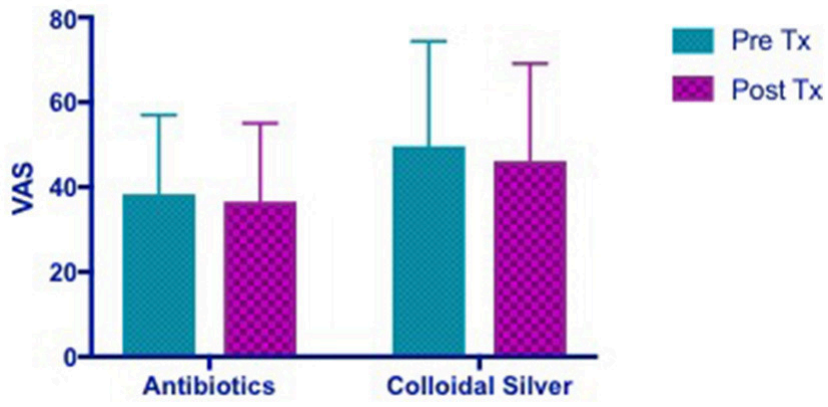
Bar graph showing no significant difference in VAS scores between CON and CS treated groups. VAS, Visual Analog Scale; CON, Control; CS, Colloidal silver.

VAS scores in both CON and CS groups showed a similar trend of improvement post-treatment, but both were not statistically significant (CON 1.728 [95% CI −7.785 to 11.24] vs. CS 3.536 [95% CI −5.977 to 13.05]) (Figure 2).

#### Sino-nasal outcome test−22 (SNOT-22)

**Figure 3:**
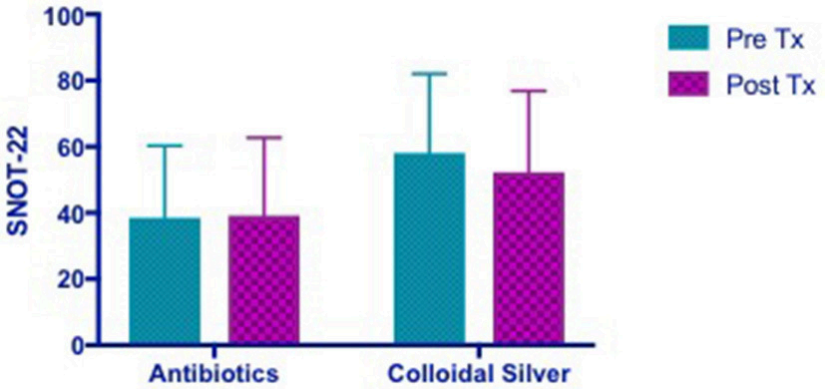
Bar graph showing no change in SNOT-22 scores in CON group, while CS group showed a trend of improved SNOT-22 scores, but not statistically significant. SNOT-22, Sino-Nasal Outcome Test-22; CON, Control; CS, Colloidal silver.

Patients in the CON group showed no change in SNOT-22 scores post- treatment while CS group showed a trend toward an improvement in SNOT-22 scores, but it was not statistically significant (CON −0.6364 [95% CI −6.673 to 5.4] vs. CS 5.818 [95% CI −0.2183 to 11.85]) (Figure 3).

#### Lund kennedy score (LKS)

**Figure 4:**
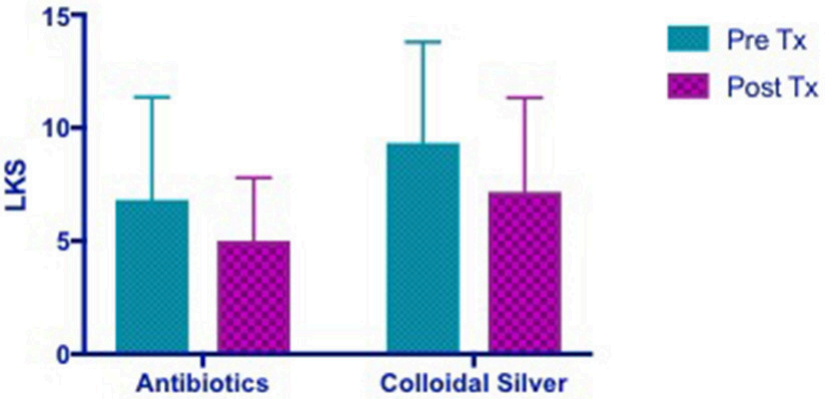
Bar graph showing similar improvements in LKS in both CON and CS groups, but not statistically significant. LKS, Lund Kennedy Scores; CON, Control; CS, Colloidal silver.

Both CON and CS group showed trends of similar improvements in Lund Kennedy Scores but this was not statistically significant (CON 1.818 [95% CI −1.373 to 5.009] vs. CS 2.167 [95% CI −2.154 to 6.488]) (Figure 4).

### Subgroup analyses: crossover silver arm

Five patients completed the crossover CS arm after failing oral antibiotics. Subgroup analyses were performed comparing VAS, SNOT-22, and LKS scores of patients while on either treatment. The mean score difference post antibiotic treatment vs. post CS treatment were compared using Wilcoxon matched-pairs signed rank tests. However, due to the small sample size of our subgroup analyses, data presented is focused on describing observed trends.

#### Microbiology result of crossover arm

1/5 patient had successful infection eradication from CS treatment after failing culture-sensitive oral antibiotics.

#### Visual analog scale (VAS) of crossover arm

**Figure 5:**
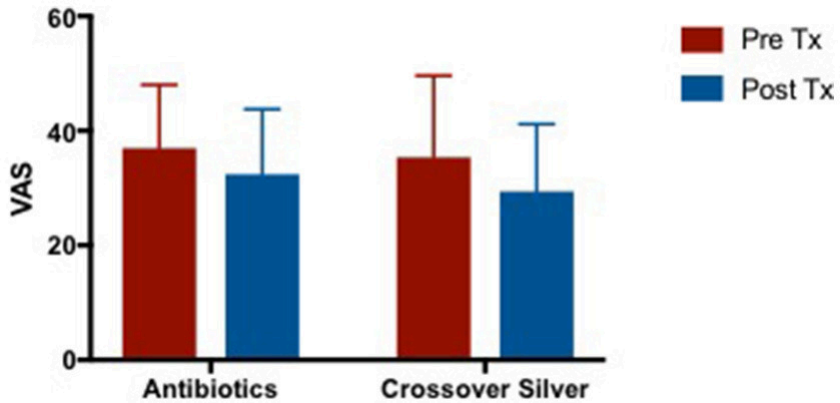
Bar graph comparing VAS scores of patients following failed culture sensitive oral antibiotics and crossed-over to CS treatment. VAS, Visual Analog Scale; CS. Colloidal silver.

There were slight improvements in VAS scores after culture sensitive oral antibiotics and CS treatment. There was a trend of greater improvement in VAS while on CS compared to when patients were treated with culture sensitive oral antibiotics. It is also observed that patients' VAS scores appeared to return to baseline after completing course of oral antibiotics and before commencing CS which is consistent with what is observed in clinical practice (Figure 5). Mean difference in VAS scores when patients were on culture sensitive oral antibiotics 4.546 [95% CI −8.156 to 17.25] vs. CS treatment 5.94 [95% CI −3.347 to 15.23], *p* = 0.4750.

#### Sino-nasal outcome test−22 (SNOT-22) of crossover arm

**Figure 6:**
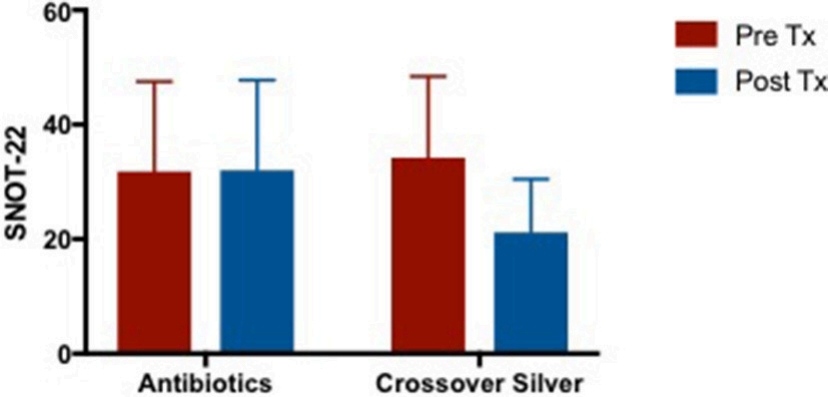
Bar graph comparing SNOT-22 scores of patients following failed culture sensitive oral antibiotics and crossed-over to CS treatment. SNOT-22, Sino-Nasal Outcome Test-22; CS, Colloidal silver.

There were no changes in SNOT-22 scores after culture sensitive oral antibiotics treatment but showed trends of improvement when patients were crossed over to CS treatment (Figure 6). Mean difference in SNOT-22 scores when patients were on culture sensitive oral antibiotics 0.2 [95% CI −2.021 to 2.421] vs. CS treatment −13 [95% CI −22.42 to −3.585], *p* = 0.06.

#### Lund kennedy score (LKS) of crossover arm

**Figure 7:**
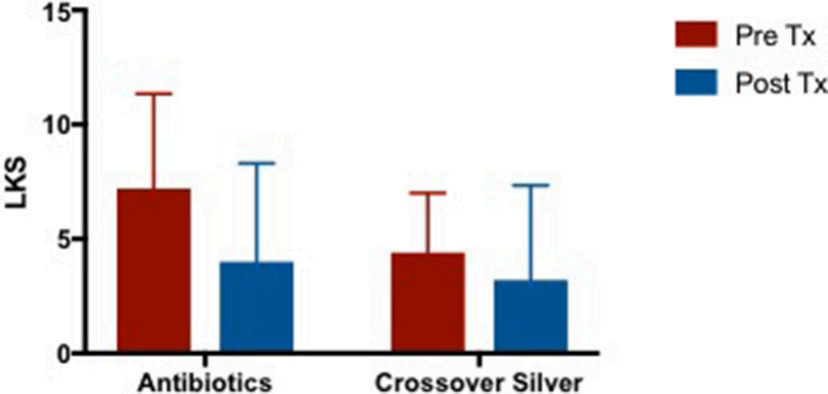
Bar graph comparing LKS scores of patients following failed culture sensitive oral antibiotics and crossed-over to CS treatment. LKS, Lund Kennedy Scores; CS, Colloidal silver.

Patients demonstrated an improvement in LKS post antibiotic treatment and further improvements were observed after completion of CS treatment (Figure 7). Mean difference in LKS scores when patients were on culture sensitive oral antibiotics −2.8 [95% CI −7.311 to 1.711] vs. CS treatment −1.4 [95% CI −4.259 to 1.459], *p* = 0.50.

### Safety

#### Serum silver levels

Four patients who had received CS had serum silver levels that were above normal limits measured within 24 h after receiving final silver dose. 3 patients had a repeat test 10 days after study exit which saw serum silver levels had returned to normal parameters. One patient had serum silver levels which were above normal ranges pre-treatment and on repeat test had returned to baseline. Our laboratory reference indicates that argyria can be present at serum silver levels of approximately 100 nmol/L, the highest level of serum silver level recorded in our study was 57.3 nmol/L.

#### Smell test

There were no significant changes in smell pre- and post-treatment between both groups measured using the University of Pennsylvania Smell Identification Test (UPSIT).

#### Adverse events

There were no serious adverse events reported.

## Discussion

In this study, looking at the primary end-point of culture negativity post-treatment, CS has not been shown to be superior to culture-directed oral antibiotics. Although interesting to note, CS patients had more severe baseline disease when compared to CON, but demonstrated comparable improvement in subjective symptoms and objective endoscopic scores suggesting it may be more than just a placebo effect. It is possible that CS treatment over 10 days is sufficient to demonstrate symptomatic and endoscopic improvement but insufficient time to achieve bacterial eradication. Indeed, when compared with topical mupirocin rinses which have been one of the more successful topical treatments for recalcitrant patients (Solares et al., 2006; Uren et al., 2008; Jervis-Bardy and Wormald, 2012; Jervis-Bardy et al., 2012; Seiberling et al., 2013), mupirocin has been used as a twice-daily rinse over 3–4 weeks. We believe that this reflects the duration of CS treatment needs to be further optimized. A longer study period including a larger number of study participants would be needed to assess the safety and efficacy of CS topical application in these patients.

The spherical CS nanoparticles used in this study has been shown to have substantial anti-biofilm activity *in vitro* with 96, 97, and 98% biofilm reduction of *S. aureus*, MRSA, and *P. aeruginosa* respectively (Richter et al., 2017c). It has been postulated that CS exerts its antimicrobial properties via multiple mechanisms. It can act on bacterial cell membranes by disrupting phosphate (Schreurs and Rosenberg, 1982) and sodium channels (Semeykina and Skulachev, 1990), inhibits mitochondrial ATPase (Chappell and Greville, 1954) and interacts with bacterial DNA to form dissociable complexes (Rosenkranz and Rosenkranz, 1972; Modak and Fox Jr., 1973).

Some immunomodulatory functions of CS have also been observed in the literature. It has the ability to inhibit matrix metalloproteinases (MMPs) which is pro-inflammatory (Wright et al., 2002) and metallothionein (Wright et al., 2002) (MT) which promotes resistance to immune-mediated apoptosis (Dutsch-Wicherek et al., 2006). Both MTs and MMPs have been found at increased levels in patient with CRS with nasal polyps (CRSwNP) (Wicherek et al., 2007; Eisenberg et al., 2008; Sauter et al., 2008). CS has also been shown to induce inflammatory cells apoptosis by TNF-α and IL-12 suppression (Bhol and Schechter, 2005). An improved host response might be able to account for the efficacy observed in the CS cohort even though there was no eradication of bacteria.

However, one of the limitations of this study is the time-consuming process of manufacturing CS rinses using small scale equipment. Currently, to prepare sufficient CS for a 10-day treatment course a full-time laboratory personnel requires over 10–15 h. If production cannot be upscaled, CS could be evaluated as an adjunct to oral antibiotics.

In the literature, silver has been described to exhibit low toxicity with minimal risks expected from clinical exposure. Silver is absorbed into the systemic circulation as a protein complex and eliminated by the liver and kidneys (Lansdown, 2006). Prolonged silver exposure commonly associated with occupational and/or systemic administration can lead to deposition of silver particles in skin (argyria), eye (argyrosis), and other organs (Tomi et al., 2004). Argyria is a cosmetic concern with irreversible blue-gray skin discoloration in sun-exposed areas, but not life-threatening.

Reported cases of silver toxicity are limited. In the literature, very little data exists correlating serum silver levels with symptomatic presentation of argyria and at present there are no medical guidelines available regarding its use. The World Health Organisation reported that a person can have a total lifetime oral intake of approximately 10 g of silver with no observed adverse effects (World Health Organisation, 1996). The United States Environmental Protection Agency's has reported that a maximum acceptable oral dose of silver to be 0.005 mg/kg/day or about 0.35 mg for a 70 kg person a day, every day during their lifetime (Fung and Bowen, 1996). In this study patients will be exposed to a total of 72 mg of topical CS rinses, which is well under the total lifetime amount of 10 g and to an equivalent of 7.2 mg/day of topical silver treatment for 10 days. Our laboratory reference of serum silver levels indicates argyria could be present when serum silver levels exceed 100 nmol/L. The serum silver levels were well below this concentration and no symptoms of argyria were observed in any patient of this study.

Although this study has shown that CS is safe based on serum silver levels and smell tests, the discomfort of using CS rinses have been noted. This discomfort is likely due to the tonicity and temperature of the rinses and possible stinging properties from silver. To improve the tonicity of the rinse solution for better tolerability, we are currently looking at mixing CS with 5% dextrose isotonic solution.

## Conclusion

This study concludes that twice daily CS (0.015 mg/mL) sinonasal rinses for 10 days is safe but not superior to culture-directed oral antibiotics. Future studies looking at optimizing the tolerability, duration of treatment and investigating the role of CS as an adjunct treatment to oral antibiotics should be explored and evaluated in a randomized, double-blinded, placebo-controlled trial.

## Author contributions

MO: project design, data collection and analysis, manuscript preparation; KR: project design, product manufacture and quality control, manuscript preparation; CB: product manufacture and quality control; LM-V: data analysis; AP project design, manuscript preparation; SV: project design, manuscript preparation; P-JW: project design, manuscript preparation.

### Conflict of interest statement

The authors declare that the research was conducted in the absence of any commercial or financial relationships that could be construed as a potential conflict of interest.

## Abbreviations

CRS: chronic rhinosinusitis
INC: intranasal corticosteroid
CON: control
CS: Colloidal Silver
VAS: Visual Analog Scale
SNOT-22: Sino-Nasal Outcome Test-22
LKS: Lund Kennedy Scores
UPSIT: University of Pennsylvania Smell Identification Test
AE: Adverse Event.
